# Current ankle sprain prevention and management strategies of netball athletes: a scoping review of the literature and comparison with best-practice recommendations

**DOI:** 10.1186/s13102-021-00342-9

**Published:** 2021-09-18

**Authors:** Patrick L. Rowe, Adam L. Bryant, Kade L. Paterson

**Affiliations:** Department of Physiotherapy, Centre for Health, Exercise and Sports Medicine, School of Health Sciences, Faculty of Medicine, Dentistry and Health Sciences, The University of Melbourne, Melbourne, VIC Australia

**Keywords:** Netball, Ankle sprains, Ankle injuries, Chronic ankle instability, External ankle support, Prevention, Management, Rehabilitation, Return to sport

## Abstract

**Background:**

Ankle sprains are the most commonly reported injury in netball. Approximately four in five netball athletes will sustain an ankle sprain, up to half will go on to sustain recurrent ankle sprains, and nine in ten report perceived ankle instability. Historically, prevention and management strategies of ankle sprains and injuries have been investigated for a variety of sports, however, no literature reviews have investigated these in netball athletes, or compared these with current best-practice within the literature. Therefore, this scoping review aims to understand how netball athletes currently prevent and manage ankle sprains and to compare these approaches with best-practice recommendations.

**Methods:**

A literature search was conducted using MEDLINE, CINAHL, and SPORTDiscus databases using keywords to capture studies with data or information related to the prevention and management of ankle sprains and injuries in netball.

**Results:**

The search strategy captured 982 studies across all databases, with 30 netball studies included in this scoping review. Studies suggest netball athletes are not commonly referred to health professionals, do not undertake adequate rehabilitation, and almost immediately return to court following an ankle sprain or injury. Current best-practices suggest injury prevention programs and external ankle support effectively reduce ankle sprains and injuries; however, poor compliance and implementation may be a significant barrier. Currently, there is a lack of evidence that netball-specific footwear reduces the risk of ankle sprains.

**Conclusion:**

The findings suggest netball athletes do not implement current best-practice prevention and management strategies following an ankle sprain. This is despite evidence of the effectiveness of injury prevention programs, external ankle support, and adequate rehabilitation in reducing ankle sprain rates. Current-best practice prevention and management of ankle sprains should be considered by clinicians, coaches, and athletes to reduce the prevalence and chronicity of ankle sprains in netball.

**Supplementary Information:**

The online version contains supplementary material available at 10.1186/s13102-021-00342-9.

## Background

Netball is one of the leading female sporting codes worldwide with over 20 million participants across 80 countries [1, 2]. It is an intermittent, high-intensity sport played within a limited court space where athletes undertake repeated cutting, pivoting, jumping, landing, and sprint efforts [3, 4]. The fast-paced, frenetic nature of netball, in conjunction with the one-step rule, is considered key factor for injury risk during match-play [5, 6]. Ankle sprains are the most commonly reported injury, accounting for approximately 40% of all netball injuries [7, 8]. In fact, netball has one of the highest incidence rates of ankle sprains in worldwide sport [7]. Ankle sprains during netball typically result from poor landing mechanics or player contact, resulting in an inversion-internal rotation mechanism [9].

One study has shown up to four in five netball athletes will have sustained at least one ankle sprain in their lifetime [10]. Unfortunately, many netball athletes sustain their index ankle sprain from a very young age, with a recent study reporting an 84% increase in the number of ankle sprains in the 10–14 year age group over 10 years [11]. Currently, there is considerable concern a large proportion of netball athletes who sustain an ankle sprain will go on to develop chronic ankle instability (CAI). CAI is characterised by recurrent ankle sprains and/or feelings or perception the ankle joint is ‘unstable’, and/or self-reported disability, for at least one year following an index ankle sprain [12, 13]. Up to half of all netball, athletes will also go on to sustain recurrent ankle sprains, more commonly bilateral recurrent sprains, following an index sprain [10, 14]. Furthermore, nine in ten netball athletes with a history of ankle sprains report some form of ankle instability, with 64% demonstrating moderate-severe instability [10].

Historically, netball injuries have been well documented since the nineteen-eighties. However, there has only been one broad review investigating injury prevention and management in netball [15]. The review did not specifically focus on ankle sprains and was published more than two decades ago. Since this time, there have been a plethora of studies have investigated injury prevention and management strategies in netball over the previous decade, in particular ankle sprains, suggesting an updated review of the literature is required [16]. To our knowledge, no study has investigated the current management and prevention strategies undertaken by netball athletes following an ankle sprain or injury, nor compared these findings with best-practice guidelines available from the literature. This scoping review aims to (1) understand how netball athletes currently prevent and manage ankle sprains or injuries, (2) compare the current practices of netball athletes with best-practice guidelines for prevention and management of ankle sprains or injuries, and (3) consider what prevention and management strategies for ankle sprains can be improved or better implemented when undertaken by netball athletes.

## Methods

### Search strategy

Due to the broad nature of the topic, a scoping review was chosen as the appropriate method of presenting the data and evidence. The preferred reporting items for systematic review and meta-analyses extension for scoping reviews (PRISMA-ScR) were adopted and are presented in Additional file 1 (PRISMA ScR checklist) [17]. A literature search was conducted using MEDLINE, CINAHL, and SPORTDiscus databases on July 15th, 2021. The search included a combination of free-text terms including “netball” AND “ankle” OR “sprain” OR “injur*” OR “instability” OR “CAI” OR “epidemiolog*” OR “incidence” OR “prevalence” OR “data” OR “statistic*” OR “pattern*” OR “rehab*” OR “treat*” OR “manage*” OR “prevent*” OR “brac*” OR “tap*” OR “ankle support” OR “footwear” OR “shoe” OR “warm-up” OR “program”. Database searches and captured studies are presented in Additional file 2 (search strategy).

### Study inclusion

Studies were eligible if they (1) were published in a peer-reviewed journal; (2) randomised, cross-sectional and observational studies explicitly investigated a netball cohort; (3) included data or information related to ankle sprains and injuries (ankle fractures, contusions, and deltoid ligament sprains); and (4) investigated prevention and management related to ankle sprains and injuries. Studies were excluded if they did not provide data or information related to ankle sprains and/or injuries or did not include a netball cohort. Non-English language studies, review articles, conference proceedings, or abstracts which did not provide sufficient data were also excluded. The reference list and citations of captured studies were cross-referenced to identify additional studies relevant to this review.

### Data extraction and analysis

Two authors (PLR and KLP) reviewed and collected data from the included studies. Author, year of publication, study design, sample size, age of participants and key findings relevant to this review were all extracted and collated. Authors of the relevant studies were contacted if data was unavailable. Following data collation, literature trends were identified and classified into sub-categories within prevention and management, and described in as a narrative synthesis. Participant data was presented as number, mean and standard deviations if available, while study outcomes were presented as proportions, ranges and p values as appropriate.

## Results and discussion

### Study identification

**Fig. 1:**
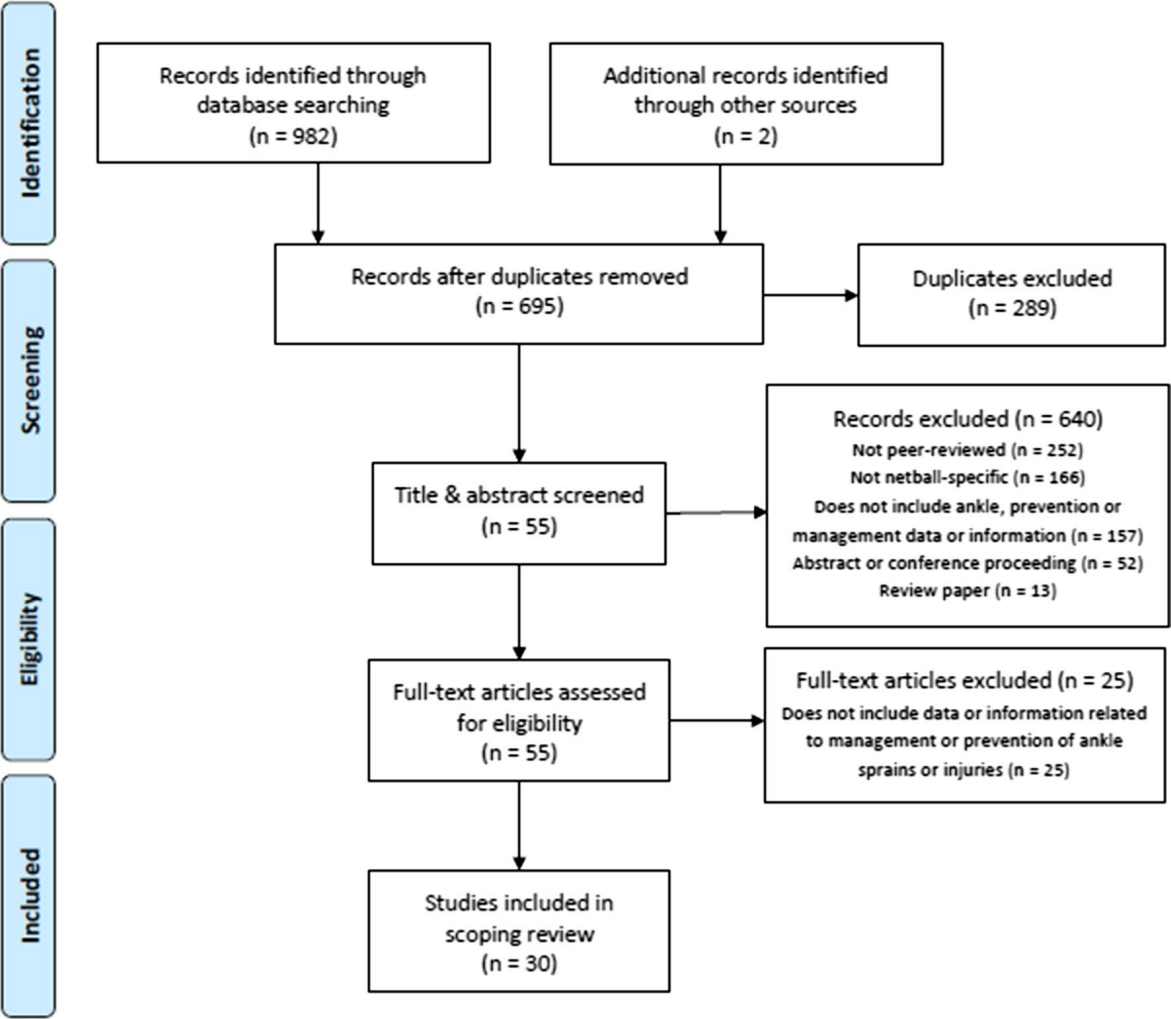
PRISMA flowchart

The search strategy captured 982 studies across all databases. Two additional studies were identified by cross-referencing and reference lists. Once duplicates were removed, 695 studies remained. Fifty-five studies remained following a review of title and abstract. Upon full-text review, thirty studies were included in this scoping review [10, 18–46]. Figure 1 provides an overview of the search strategy and study inclusion using the PRISMA flowchart.

### Prevention of ankle sprains in netball

Twenty-five studies presented data related to the prevention of ankle sprains and injuries in netball. Three subcategories were identified; these include injury prevention programs (14 studies), external ankle support (11 studies), and footwear (5 studies).

#### Injury prevention program

In South Africa, more than half of all injured elite netball athletes reportedly did not undertake core stability, proprioceptive, or neuromuscular and landing training [27]. One study found that a six-week gluteal strengthening, core stability, and proprioceptive program improved dynamic balance in university netball athletes [33]. In 2013, Netball New Zealand introduced a dynamic warm-up and education program titled NetballSmart [29]. Two years later, Netball Australia implemented a nationwide injury prevention program titled Knee Injury Prevention for Netballers to Enhance Performance and Extend Play (KNEE) aiming to reduce lower limb injuries, in particular knee and ankle injuries [26]. The NetballSmart program has been shown to reduce peak vGRF and improve landing mechanics in junior netball athletes [34]. But to this date, no study has investigated the effectiveness of the NetballSmart and KNEE programs on ankle injury rates in netball.

Currently, the greatest challenge of injury prevention programs in netball is poor implementation rates [41, 42]. Only 12–18% of the recommended activities from the Netball KNEE program were undertaken by community-level junior netball athletes [40]. Concerningly, strength, balance, and agility-specific exercises were rarely performed [40]. Evidence suggests that trunk and lower limb strengthening and proprioceptive exercises significantly reduce injury rates, particularly ankle injuries, whilst also improving sprint, agility, and jumping performance [35, 37, 39]. Poor implementation of injury prevention programs at the community level may limit its influence on netball injuries [40]. Barriers include athlete and coach engagement, education, resources, and time [41, 42]. Education sessions for netball coaches resulted in greater knowledge and implementation of injury prevention programs [36]. The authors recommended the inclusion of coach education sessions and accessible resources to improve implementation rates [36]. Optimistically, more than four in five netball coaches strongly support the use of injury prevention programs and report competency in teaching a safe landing program to junior netball athletes [44]. Furthermore, most netball athletes report positive beliefs and attitudes towards undertaking a safe landing program [45].

#### External ankle support

External ankle support is a common injury prevention measure undertaken by netball athletes. One study found 34.4% of state netball athletes wore external ankle support during a tournament [23]. Another study found approximately half of netball athletes reported using taping (30.8%) or bracing (18.8%) [24]. However, the same study found 68.2% were not wearing external ankle support when they sustained an ankle injury [24]. Attenborough et al. [10] found 70% of club and inter-district netball athletes with CAI regularly use external ankle support, suggesting the implementation of external ankle support dramatically increases once an ankle sprain is sustained. Interestingly, one study reported a three-fold increase in lower limb injury risk for netball athletes who wore external ankle support but did not provide data specifically on ankle sprains [46].

Several studies reported significant reductions in sagittal or frontal plane biomechanics with external ankle support during landing and cutting tasks [25, 30, 31]. Furthermore, significant reductions in gastrocnemius and peroneus longus electromyography (EMG) activity were also shown with the addition of external ankle support during landing tasks [28]. With external ankle support, time to peak ground reaction forces (GRF) was reduced, but had no influence on peak GRF and ankle joint moments during side-stepping and landing tasks [25, 28]. Proprioception, measured using active movement extent discrimination apparatus (AMEDA), was improved with self-applied taping (0.022) and taping administered by a health professional (0.034) compared to no external ankle support, but neither was more efficacious than the other [43]. Currently, the NetballSmart and KNEE injury prevention programs do not endorse the use of external ankle support for the prevention of ankle injuries [26, 29].

#### Footwear characteristics

Early studies investigated the popularity and influence of shoe collar height in netball, with three collar heights (low-, mid-, and high-cut) commonly reported. One study found 60.0% of elite Jamaican netball athletes wore mid-cut footwear, less than half wore low-cut footwear (37.2%) and very few wore high-cut footwear (2.7%) [18]. In a second study, more than half (54.9%) of state-netball athletes were wearing mid-cut footwear and 35.7% were wearing low-cut footwear when they sustained an ankle injury [24]. Given these studies were cross-sectional, however, it is unclear whether shoe collar height influences the risk of sustaining an ankle sprain in netball. A third study found no association between the age of netball shoes and lower limb injuries [46]. Biomechanical studies have reported that ankle kinematics, peak GRF, and ankle joint moments were not influenced by netball-specific footwear during side-stepping tasks [25]. However, netball-specific footwear has been reported to increase time to peak impact and reduced loading rates during running, cutting, and landing tasks, suggesting it may have the potential to reduce injuries in netball [32].

### Management of ankle sprains in netball

Five studies presented data related to the management of ankle sprains and injuries in netball. Three subcategories were identified; these include treatment and rehabilitation (3 studies) and return to sport (3 studies).

#### Treatment and rehabilitation

The literature suggests that a minority of netball athletes who sustain an ankle sprain or injury are referred to a health professional. Two studies reported extremely low referral rates (14.1–27.1%) [21, 22], while another study reported higher referral rates to a physiotherapist (76.9%) [20]. Composite treatment was most commonly undertaken by community-level netball athletes following an ankle injury (66.3–68.8%) [21, 22]. Injury advice and home exercise programs were commonly provided (56.9–77.7%). Ice and rest, in isolation or combination, were prescribed less often (0.0–31.4%) [21, 22]. No study investigated the types of rehabilitation exercises undertaken by netball athletes following an ankle sprain or injury.

#### Return to sport

Early return to sport following ankle sprains and injuries were consistently reported across all studies in netball. During an international netball tournament, ankle sprains accounted for 17.4% of all injuries, with a quarter resulting in time-loss between 1–7 days (12.5%) and 8–28 days (12.5%) [38]. A second study reported three in four state-netball athletes returned to court immediately following an ankle sprain during a netball tournament [19]. Hopper et al. [20] reported 38.5% of community netball athletes returned to court for the following game during a 14-week season [20]. Of these, more than a third of netball athletes did not miss a netball match (38.5%), 15.4% missed one match, 38.5% missed two matches and very few missed three or more games (7.7%) [20]. No study reported whether netball athletes undertook to return to sport testing and/or received medical clearance before returning to netball (Table 1).

**Table 1:** Study characteristics of included studies

Author	Design	Participants & Study Purpose	Condition	Results
Antcliff [26]	Expert Opinion	In 2015, Netball Australia introduced the KNEE injury prevention program to reduce the rate of lower limb injuries in netball, in particular knee and ankle injuries,	IPP	No results were specified
Attenborough [10]	Cross-Sectional	96 club and interdistrict netball athletes (24.1 ± 7.9y) undertook questionnaires to investigate the prevalence of ankle sprains, perceived and mechanical instability	EAS	70% of netball athletes with CAI regularly used external ankle support when participating in netball
Barnes [33]	RCT	16 university netball athletes (19.0y) were randomly allocated to a 6-week PROP (n = 8) and CONT (n = 8) to compare the effects of proprioceptive training on dynamic balance	IPP	PROP group demonstrated a statistically significant improvement in ANT, PMED, and PLAT direction of SEBT (p < 0.05)
Belcher [34]	RCT	77 junior netball athletes (15.9 ± 0.9y) were randomly allocated to a 12-week NSDW (n = 37) or PWU (n = 40) to compare the effects on peak force and landing performance	IPP	NSDW and PWU groups significantly reduced peak vGRF and LESS (p < 0.05). PWU had a significantly greater improvement in LESS compared to NSDW (p = 0.001)
Coetzee [27]	Cross-Sectional	1,280 elite netball athletes (age NS) undertook questionnaires and injury surveillance over a 4–6 day tournament to determine incidence rates and the influence of training habits	IPP	70 ankle injuries were reported (34% prevalence). More than half of injured netball athletes did not undertake core stability (51.7%), proprioceptive (59.0%), or neuromuscular and landing training (57.7%),
Elphinston and Hardman [35]	Observational	17 international netball athletes (age 25.9 ± 2.6y) with inexperience in detailed training prescription undertook injury surveillance over 2-years to determine the effects of a functional stability program	IPP	A significant reduction in ankle injuries (six to one) was observed between two calendar years
Franettovich-Smith et al. [46]	Prospective Cohort	269 community netball athletes (15.0 ± 5.0y) undertook questionnaires and injury surveillance over one season to determine incidence rates, mechanism, and predictor of injuries	EAS NSF	44 ankle injuries were reported (26% prevalence). Netball athletes who implemented external ankle support had a three-fold increase in sustaining a lower limb injury compared to netball athletes who do not use taping or bracing (P < 0.001). No association was found between the age of netball shoes and lower limb injuries (p = 0.261)
Gianotti et al. [36]	Cross-Sectional	217 netball coaches (age NS) completed a survey following an NSDW education and resources course to assess its effectiveness on implementation	IPP	Most netball coaches read the NSDW booklet (79%), changed the way they coached (89%), used information from injury prevention programs (94%), and passed it on to their athletes (90%). 70% reported changes to their player’s landing, stopping, dodging techniques, and recovery procedures
Greene et al. [25]	Case-Crossover	10 elite netball athletes (18.3 ± 1.9y) completed a side-step cutting task to compare the effects of NSF, NSF + LUB, and HTF on ankle biomechanics	EAS NSF	NSF + LUB recorded a significant reduction in SAG ankle excursion compared to NSF (p < 0.05). No difference was found between NSF, NSF + LUB, and HTF in SAG and FRO ankle excursion, moments, and GRF
Hopper [22]	Prospective Cohort	3,108 community netball athletes (age NS) undertook questionnaires and injury surveillance over a 14-week season to determine incidence rates and treatment strategies	T&R	92 ankle injuries were reported (57% prevalence). 66.3% received composite treatment and 33.7% were advised ice. 77.7% received advice. None were advised to rest. 14.1% were referred to a doctor or physiotherapist
Hopper and Elliott [23]	Prospective Cohort	228 state netball athletes (21.4 ± 3.7y) undertook questionnaires and injury surveillance over a 9-day tournament to determine incidence rates and characteristics of injuries	EAS	19 ankle injuries were reported (37% prevalence). 34.4% of netball athletes reported were wearing tape or brace when playing netball
Hopper et al. [20]	Prospective Cohort	11,228 community netball athletes (age NS) undertook questionnaires and injury surveillance over a 14-week season across 5 years to determine incidence rates and treatment strategies	T&R	513 ankle/foot injuries were reported (84% prevalence). 68.8% received composite treatment and 31.4% were advised ice and rest following an ankle/foot injury. 56.9% received advice and a home exercise program. 27.1% were referred to a physician or physiotherapist
Hopper et al. [21]	Prospective Cohort	72 community netball athletes (age NS) undertook questionnaires and injury surveillance over a 14-week season to determine incidence rates and treatment strategies	T&R RTS	13 ankle sprains were reported (59% prevalence). 76.9% were referred to a doctor or physiotherapist and received treatment. None returned to the following training session. All players missed at least one (46.8%), two (46.1%), and three (7.7%) training sessions. Most players missed zero (38.5%) one (15.8%) and two matches (38.5%). Very few missed three matches (7.7%)
Hopper et al. [28]	Case-Crossover	15 elite netball athletes (22.6 ± 4.2y) completed a jump-land task to compare the effects of LUB, NET, and BF on ankle biomechanics	EAS	LUB significantly reduced EMG activity of gastrocnemius and peroneal longus muscles compared to NET and BF (p < 0.007). No difference was found in peak vGRF and TTP between LUB, NET, and BF
Hopper et al. [37]	RCT	23 junior netball athletes (12.2 ± 0.9y) were randomly allocated to a 6-week NMT (n = 13) or CONT (n = 10) to compare effects on ankle biomechanics	IPP	NMT group significantly improved 10 m sprint, 20 m sprint, 505 agility, CMJ height, and peak power, NMST score and ANT, PMED, and PLAT directions of SEBT (p < 0.05)
Hume and Steele [24]	Prospective Cohort	940 representative netball athletes undertook questionnaires and injury surveillance over a 3-day tournament to determine incidence rates and characteristics of injury	EAS NSF	44 ankle injuries were reported (14%). Approximately half of all netball athletes used taping (30.8%) or bracing (18.8%) during netball. Of the ankle injuries, 68.2% reported not wearing EAS, 54.9% wore mid-cut, and 35.7% sore low-cut footwear (35.7%). Very few wore high-cut footwear (9.5%)
Janse van Rensburg et al. [38]	Prospective Cohort	192 international netball athletes undertook injury surveillance over a 10-day tournament to determine incidence rates and characteristics of injury	RTS	8 ankle sprains were reported (17% prevalence). 75.0% returned to netball immediately following an ankle sprain. Ankle sprains resulting in time-loss were between 1–7 days (12.5%) and 8–28 days (12.5%)
Kearney [29]	Expert Opinion	In 2013, Netball New Zealand introduced the NSDW injury prevention program to reduce the rate of lower limb injuries in netball, in the particular knee and ankle injuries,	IPP	No results were specified
Masharawi etal. [30]	Case-Crossover	10 elite netball athletes completed a weight-bearing inversion test using an SRB and LUB to compare the effect on ankle kinematics	EAS	SRB and LUB significantly reduced ankle inversion angle before and after exercise, compared to no bracing (p < 0.001). No difference was found between SRB and LUB
Mason-Mackay et al. [31]	Case-Crossover	20 high school netball athletes completed drop-jump, drop-land, and netball-jump tasks with LUB and NS to compare the effect on ankle biomechanics and balance	EAS	LUB significantly reduced SAG ankle excursion during drop-jump, drop-land, and netball-jump tasks (p < 0.10). LUB increased ankle stiffness during drop-lands (p < 0.10). No difference was found in peak vGRF and TTP
Mckenzie et al. [39]	RCT	81 youth netball athletes were randomly allocated to NSDW (n = 45) and TWU (n = 36) to compare the effects on performance measures	IPP	NSDW group recorded significant improvements in prone hold (p = 0.01), vertical jump (p = 0.01) and reduction in horizontal jump performance (p = 0.03)
Saad et al. [40]	Observational	66 community netball coaches were observed conducting 67 team training sessions across 4 clubs to assess implementation rates of the KNEE program	IPP	Implementation of the netball KNEE program was low in the 7–10-year (12%), 11–13-year (18%), and 14 + year (14%) age groups. 28% of teams completed warm-up and footwork exercises. Strength, balance, and agility-specific exercises were rarely performed
Saunders et al. [41]	Cross-Sectional	31 junior netball coaches completed a one-hour workshop and implemented a 6-week injury prevention program at team training sessions and completed a survey to assess benefits and barriers	IPP	Coaches reported subjective improvements in player’s athletic attributes (83%), landing technique (79%), and reduced knee and ankle injury risk (79%). Perceived coaching barriers included lack of player motivation (83%), ideas for training drills (79%), non-attendance (71%), and time (63%)
Sinclair et al. [32]	Case-Crossover	12 university netball athletes completed running, cutting, and vertical jumping tasks with NSF and MF to compare the effects on ankle biomechanics	NSF	MS significantly decreased time to peak loading and increased loading rate during running, cutting, and vertical jumping tasks (p < 0.05). MS significantly increased peak eversion angle during running (p < 0.05). No difference was found in SAG, FRO, and TRA ankle IC, excursion, and peak angles
Singh et al. [18]	Cross-Sectional	59 elite netball athletes (age NS) undertook questionnaires and injury surveillance during tournaments across 5 years to determine incidence rates and characteristics of injury	NSF	24 ankle sprains were reported (56% prevalence). Most netball athletes wore medium-cut footwear (60%). Low-cut footwear (37.3%) was more commonly worn than high-cut footwear (2.7%)
Smyth et al. [19]	Prospective Cohort	103 state netball athletes (U17 and U19) undertook questionnaires and injury surveillance during a 6-day tournament to determine incidence rates and characteristics of injury	RTS	14 ankle sprains were reported (14% prevalence). 28.6% resulted in time-loss following injury (no time specified)
Smyth et al. [42]	Mixed-Methods	39 state-team netball coaches, strength and conditioning coaches, and physiotherapists discussed the challenges associated with the implementation of the KNEE program	IPP	Eight common themes were identified including athlete (engagement and technique), staff (resourcing, prioritization, and supervision), and program (education, time constraints, flexibility, and adaptability) barriers
Smyth et al. [43]	RCT	53 sub-elite netball athletes completed single leg balance on an inversion tilt platform with S-NET (n = 26), HP-NET (n = 27) pre-and post-training to compare the effects on proprioception	EAS	Significant improvement in proprioception (AMEDA scores) with S-NET (p = 0.05) and HP-NET (p < 0.01). No significant difference was found between S-NET and HP-NET (p = 0.90)
White et al. [45]	Cross-Sectional	287 junior netball athletes completed a questionnaire on the attitudes, social norms, behaviour, and intention to learn a safe landing program	IPP	A high number of netball athletes reported learning a safe landing program being extremely helpful (54.4%), useful (57.8%), and good (46.7%). Netball athletes reported it would be extremely possible (39.0%), Netball athletes also felt that it was extremely possible (39.0%) that they could learn (41.1%) and intend on completing the program (32.1%) for every training session
White et al. [44]	Cross-Sectional	51 junior netball coaches completed a survey on the competency and benefits of teaching a safe landing program to their netball athletes	IPP	Most netball coaches reported being extremely positive a safe landing program would be beneficial (78%), valuable (71%), and positive teaching (59%). Fewer netball coaches felt extremely positive about their capabilities (31%), ability (47%), and having complete control (47%)

*AMEDA* active movement extent discrimination apparatus, *ANT* anterior, *BF* barefoot, *CAI* chronic ankle instability, *CONT* control group, *EAS* external ankle support, *FRO* frontal plane, *GRF* ground reaction force, *HP-NET* health professional applied non-elastic taping, *HTF* high-top footwear, *IC* initial contact, *IPP* injury prevention program, *KNEE* knee injury prevention for netballers to enhance performance and extend play, *LESS* landing error scoring system, *LUB* lace-up bracing, *MF* minimalist footwear, *NET* non-elastic taping, *NMT* neuromuscular training, *NS* not specified, *NSDW* NetballSmart Dynamic Warm-Up, *NSF* netball-specific footwear, *PLAT* posterolateral, *PMED* posteromedial, *PROP* proprioceptive training, *PWU* power warm-up, *RTS* return to sport, *SAG* sagittal plane, *SEBT* star excursion balance test, *S-NET* self-applied non-elastic taping, *T&R* treatment and rehabilitation, *TRA* transverse plane, *TTP* time to peak force, *TWU* traditional warm-up, *vGRF* vertical ground reaction force

### Best-practice prevention of ankle sprains

The following section describes the current best practice prevention for ankle sprains within the literature. These findings and prevention strategies undertaken by netball athletes described previously are then presented in Table 2.

**Table 2:** Current prevention practices of ankle sprains in netball compared to best practice recommendations

	Netball	Best-practice recommendations
Injury Prevention Program	The NetballSmart and KNEE programs are currently endorsed by netball-governing bodies to reduce lower limb injuries, including ankle sprains. There is evidence that neuromuscular, proprioceptive, and lower limb strengthening improves proprioception, dynamic balance, and landing mechanics in netball athletes. However, poor implementation of injury prevention programs in netball may limit their effectiveness	The evidence suggests injury prevention programs are highly effective in reducing ankle sprain rates in many sports. The early signs of the netball-specific program are promising, however, further data is required to determine their effectiveness and implementation at all competition levels. This may be of benefit for NetballSmart and KNEE programs at the community level to improve adoption and implementation rates
External Ankle Support	Less than half of netball athletes wear external ankle support during netball. Two-in-three did not have external ankle support implemented when they sustained an ankle injury. Higher implementation rates are shown in netball athletes with CAI. External ankle support restricts sagittal and frontal plane kinematics but has minimal influence on kinetics. External ankle support is currently not endorsed by netball-specific injury prevention programs	There is strong evidence that taping and bracing reduces the rate of ankle sprains. As both types of external ankle support are effective, netball athletes may preferentially choose between taping and bracing. There may also be some merit for netball-governing bodies to include external ankle support as a recommendation within their injury prevention programs to improve implementation rates
Footwear	Mid-cut footwear is most commonly worn by netball athletes. Most were wearing mid-and low-cut footwear during ankle injuries. Netball-specific footwear has minimal influence on ankle kinematics but increases time to peak impact and reduce loading which may have the potential to reduce netball injuries	There is inconclusive evidence that specific types of footwear reduce the rates of an ankle sprain. Further studies are necessary for determining whether footwear types may influence ankle biomechanics during netball-specific tasks that predispose netball athletes to sustain an ankle sprain

*CAI* chronic ankle instability, *KNEE* knee injury prevention for netballers to enhance performance and extend play

#### Injury prevention programs

Injury prevention programs are a multi-modal combination of training strategies aiming to enhance strength, balance, landing, agility, and sport-specific tasks [47]. Single limb proprioceptive and neuromuscular exercises incorporating perturbation or sport-specific tasks have been shown to reduce ankle injuries by 30–45% [47] (Table 2). More recent netball studies have shown a 5–15% reduction in ankle injuries in New Zealand since the inception of the NetballSmart program [11]. Similar to NetballSmart and KNEE programs, other sporting codes across the world have implemented injury prevention programs including the Fédération Internationale de Football Association (FIFA) 11 + , FootyFirst, and Prep-to-Play programs [48–50]. The FIFA 11 + has been shown to significantly reduce ankle sprain rates and severity of injury [51, 52]. Despite their effectiveness, low implementation rates of injury prevention programs within community sport remain a significant issue [53]. To address this, Australian Football has established implementation planning for FootyFirst to promote the adoption, resources, and effectiveness of injury prevention programs at the community level [54]. This may be of benefit for NetballSmart and KNEE programs at the community level to improve adoption and implementation rates. Overall, the evidence suggests injury prevention programs are highly effective in reducing ankle sprain rates in many sports. The early signs of the netball-specific program are promising, however, further data is required to determine their effectiveness and implementation at all competition levels (Table 2).

#### External ankle support

Evidence supports the use of external ankle support to effectively reduce ankle sprains [47, 55]. In particular, taping and bracing were extremely effective in reducing secondary ankle sprains [56] (Table 2). Regarding primary prevention, low-quality studies and significant heterogeneity make it difficult to determine the effectiveness of external ankle support [55, 56]. There is no clear indication of whether taping or bracing was more efficacious [56]. Bracing is often preferred over taping due to its simplicity, ease of application, and re-usable nature making it more practical and cost-effective [57]. In contrast, some athletes may prefer taping as it may provide greater comfort, support, compliance, and variability [58]. Despite it's effectiveness, netball athletes generally do not implement external ankle support until they have sustained an ankle sprain or developed instability [10, 23, 24]. Therefore, we recommend the use of taping or bracing to reduce the risk of ankle sprains during netball participation (Table 2). As both types of external ankle support are effective, netball athletes may preferentially choose between taping and bracing. There may also be some merit for netball-governing bodies to include external ankle support as a recommendation within their injury prevention programs to improve implementation rates [26, 29].

#### Footwear

There is currently no evidence that sport-specific footwear effectively reduces ankle sprain rates [59, 60]. A recent systematic review found very few studies have investigated footwear type and its effect on ankle sprains, and the studies have shown no effect on reducing primary and secondary ankle sprains [56]. A prospective study also found shoe design did not influence the incidence of ankle sprains [61]. Due to inconclusive and limited evidence, we cannot conclusively recommend certain types of footwear in reducing the risk of ankle sprains. Further studies are necessary for determining whether footwear style, such as netball-specific footwear, may influence ankle biomechanics during netball-specific tasks that may predispose netball athletes to sustain an ankle sprain (Table 2).

### Best-practice management of ankle sprains

The following section describes the current best practice management for ankle sprains within the literature. These findings and management strategies are undertaken by netball athletes described previously are then presented in Table 3.

**Table 3:** Current management practices of ankle sprains in netball compared to best practice recommendations

	Netball	best-practice recommendations
Treatment and rehabilitation	The evidence suggests a minority of netball athletes who sustain an ankle sprain or injury are referred to a health professional. Composite treatment was most commonly undertaken by community-level netball athletes following an ankle injury. Injury advice and home exercise programs were commonly provided. Ice and rest, in isolation or combination, were prescribed less often	Rehabilitation-Oriented Assessment (ROAST) [68] (1) Self-reported pain (NPS or FADI); (2) Ankle joint swelling (FEM); (3) Ankle ROM (WBLT or A-SEBT); (4) Talocrural joint arthrokinematics (PTGT); (5) Muscle strength (hand-held dynamometry); (6) Static postural balance (BESS or FLT); (7) Dynamic postural balance (SEBT); (8) Gait (Visual Assessment); (9) Pre-injury physical activity level (Tegner Scale); (10) Patient-reported outcome measures (FADI or FAAM)
		Perceptual-Interdependence Framework [69] NICE – NSAIDs, ice, compression, and elevation EASY—external ankle support for at least 12 months following injury Optimal Loading—early commencement of ankle–foot mobilisation Exercise Rehabilitation—balance and coordination exercise program
Return to sport	Early return to sport following ankle sprains and injuries were consistently reported across all studies in netball. Up to three-in-four netball athletes returned to court immediately following an ankle sprain. Time-loss following an ankle sprain varied across studies. All netball athletes were able to return to court within 4 weeks of their injury. Most were able to return within one or two matches following their ankle sprain. No studies reported whether netball athletes undertook to return to sport testing and/or received medical clearance before returning to netball	PAASS Framework [73] (P) Pain severity (during sports participation and over the last 24 h); (A) Ankle impairments (ROM, strength, endurance and power); (A) Athlete perception (perceived confidence/reassurance, stability & psychological readiness); (S) Sensorimotor control (proprioception & dynamic postural control/balance; (S) Sport/functional performance (hopping, jumping, agility, sport-specific activities & ability to complete a full training session)

*A-SEBT* anterior reach of Star Excursion Balance Test, *BESS* Balance Error Scoring System, *FAAM* Foot and Ankle Ability Measure, *FADI* Foot and Ankle Disability Index, *FEM* figure of eight measurement, *FIFA* Fédération Internationale de Football Association, *HHD* hand-held dynamometry, *NPS* numerical pain scale, *PTGT* Posterior Talar Glide Test, *ROM* range of motion, *SEBT* star excursion balance test, *WBLT* weight-bearing lunge test

#### Treatment and rehabilitation

Generally, there is a misconception that ankle sprains are “simple” injuries, which may result in poor rehabilitation and premature return to sport [62, 63]. Similar to netball, research in other sports have shown many individuals do not seek medical advice and/or treatment from a health professional following an ankle sprain [64, 65]. A study by Hubbard-Turner [64] found two in three university students with CAI did not receive medical treatment following an ankle sprain, resulting in higher recurrent sprains, instability, and lower self-reported function. Using this evidence, poor medical-seeking behaviour and inadequate rehabilitation by netball athletes following an ankle sprain may contribute to high rates of recurrent ankle sprains and perceived instability previously reported in the literature [10, 14]. This emphasises the importance of good quality management following an ankle sprain to reduce the risk of developing CAI. In netball, insurance data shows a disproportionate number of ankle sprains and injuries receiving claims (29.3–31.0%) [66, 67], compared to ankle injury rates (40%) reported in epidemiological studies [7]. This may be due to a large number of ankle sprains or injuries that may not have been captured as some netball athletes may have continued participation, didn’t seek medical treatment, were unsuccessful, or did not complete an insurance claim [66].The rehabilitation-oriented assessment (ROAST) was developed in 2018 by the International Ankle Consortium (IAC) and is considered one of the leading assessment tools for acute ankle sprains [68]. The ROAST is comprised of ten assessment measures to identify physical and psychological impairments presenting following an ankle sprain which can be addressed during rehabilitation (Table 3) [68]. In 2019, McKeon and Donovan [69] published a clinical commentary on the conservative management of ankle sprain using a perceptual-interdependence framework. Four best-practice recommendations were considered for the effective rehabilitation of ankle sprains, with the aim of re-establishing normal function, cell-tissue-body connection and sensory-motor function to the ankle–foot complex to reduce the negative sequelae associated with ankle sprains. We recommend clinicians incorporate the PAASS framework into their decision-making process when determining safe return to play for netball athletes following an ankle sprain (Table 3).

#### Return to sport

Until recently, there had been no consensus or criteria for a safe return to sport following an ankle sprain for any sport. Very few studies clearly define return to sport criteria following an ankle sprain, however, assessment of ankle range of motion, strength, neuromuscular control, balance, psychological readiness, and sport-specific tasks were common trends [70–72]. The lack of consensus demonstrates the current challenges clinicians face when determining the athlete’s readiness to return to sport and may reflect the present attitudes and beliefs surrounding premature return to sport with ankle sprains. In netball, a large proportion of athletes return to sport almost immediately following an ankle sprain [19, 20, 38]. However, more research is required to determine if these athletes seek medical advice, undertake rehabilitation, and/or complete return to sport testing. In 2021, Smith et al. [73] undertook a Delphi study comprising of 155 health professionals to establish a consensus on assessment items determining appropriate return to sport following a lateral ankle sprain. The PAASS framework was developed comprising of five domains, including; pain severity, ankle impairments, athlete perception, sensorimotor control, and sport/functional performance [73]. The PAASS framework aims to improve assessment and decision-making for return to sport following a lateral ankle sprain [73]. We recommend clinicians incorporate the PAASS framework into their decision-making process when determining safe return to play for netball athletes following an ankle sprain (Table 3).

### Limitations and future research

There is a need for more research examining the primary prevention and management of ankle sprains and injuries in netball athletes. In comparison to knee or ACL injuries, there are limited studies investigating the prevention and management of ankle sprains. Crucially, very few studies have investigated the management trends of ankle sprains in netball and the consequences of insufficient rehabilitation, leading to the development of CAI. With the recent implementation of injury prevention programs by netball governing bodies, further research should determine the effectiveness of these programs and their specific modalities in reducing ankle sprains. Additional research is also needed to determine the effects of netball-specific footwear, in isolation and in combination with external ankle support, on ankle sprains, instability, and lower limb injuries. Finally, the best-practice recommendations included within this review are generic guidelines within the literature, and not netball-specific, which may limit its translation from research to practice.

## Conclusion

The findings of this scoping review suggest netball athletes do not implement current best-practice prevention and management strategies following an ankle sprain. Best-practice management includes a comprehensive rehabilitation and return to sport criteria following an ankle sprain, but the evidence shows netball athletes are not commonly referred to health professionals and almost immediately return to court. Netball-governing bodies currently endorse the use of injury prevention programs, but further studies are required to determine their effectiveness in preventing ankle sprains. Evidence suggests external ankle support influences ankle biomechanics and reduces the risk of ankle sprains, but netball athletes were only more likely to implement external ankle support after sustaining an ankle injury or developing CAI. Netball-specific footwear may be useful for reducing overuse injuries, however, there is a lack of evidence to suggest that specific footwear reduces the risk of ankle sprains. Current-best practice prevention and management of ankle sprains should be considered by clinicians, coaches, and athletes to reduce the prevalence and chronicity of ankle sprains in netball.

## Supplementary Information

**Additional file 1**. PRISMA-ScR checklist.

**Additional file 2**. Search strategy.

## Data Availability

All data generated or analysed during this study are included in this published article.

## Abbreviations

CAI: Chronic ankle instability
IAC: International Ankle Consortium
EMG: Electromyography
ROAST: Rehabilitation-oriented assessment
ROM: Range of motion
RTS: Return to sport
