# Supplementary material for: Current ankle sprain prevention and management strategies of netball athletes: a scoping review of the literature and comparison with best-practice recommendations
Source: BMC Sports Sci Med Rehabil. 2021 Sep 18;13:113. doi: 10.1186/s13102-021-00342-9 (PMC8449445; doi:10.1186/s13102-021-00342-9)
Supplement: Supplementary file 1 — Additional file 1. PRISMA-ScR checklist. [file 13102_2021_342_MOESM1_ESM.docx]

Supplementary file 1: PRISMA-ScR checklist
