# Supplementary material for: Current ankle sprain prevention and management strategies of netball athletes: a scoping review of the literature and comparison with best-practice recommendations
Source: BMC Sports Sci Med Rehabil. 2021 Sep 18;13:113. doi: 10.1186/s13102-021-00342-9 (PMC8449445; doi:10.1186/s13102-021-00342-9)
Supplement: Supplementary file 2 — Additional file 2. Search strategy. [file 13102_2021_342_MOESM2_ESM.docx]

Supplementary file 2: search strategy

| Step | Search terms | CINAHL results | MEDLINE results | SportsDiscus results |
| --- | --- | --- | --- | --- |
| S1 | netball | 182 | 256 | 1,503 |
| S2 | ankle OR sprain OR injur* OR instability OR CAI OR epidemiolog* OR incidence OR prevalence OR data OR statistic* OR pattern* | 2,490,259 | 10,118,092 | 445,861 |
| S3 | rehab* OR treat* OR manage* OR prevent* OR warm up OR program OR brac* OR tap* OR ankle support OR footwear OR shoe | 2,812,965 | 10,268,124 | 623,849 |
| S4 | S2 OR S3 | 4,082,917 | 16,170,114 | 856,351 |
| S5 | S1 AND S4 | 148 | 198 | 636 |
